# Multiple epigenetic factors co-localize with HMGN proteins in A-compartment chromatin

**DOI:** 10.1186/s13072-022-00457-4

**Published:** 2022-06-27

**Authors:** Bing He, Iris Zhu, Yuri Postnikov, Takashi Furusawa, Lisa Jenkins, Ravikanth Nanduri, Michael Bustin, David Landsman

**Affiliations:** 1 Protein Section, Laboratory of Metabolism, Center for Cancer Research, National Cancer Institute, National Institutes of Health, Bethesda, MD 20892 USA; 2 Computational Biology Branch, National Center for Biotechnology Information, Intramural Research Program, National Library of Medicine, Bethesda, MD 20894 USA; 3 Laboratory of Cell Biology, Center for Cancer Research, National Cancer Institute, National Institutes of Health, Bethesda, MD 20892 USA

**Keywords:** HMGN, Chromatin structure, Hi–C, Mass spectrometry

## Abstract

**Background:**

Nucleosomal binding proteins, HMGN, is a family of chromatin architectural proteins that are expressed in all vertebrate nuclei. Although previous studies have discovered that HMGN proteins have important roles in gene regulation and chromatin accessibility, whether and how HMGN proteins affect higher order chromatin status remains unknown.

**Results:**

We examined the roles that HMGN1 and HMGN2 proteins play in higher order chromatin structures in three different cell types. We interrogated data generated in situ, using several techniques, including Hi–C, Promoter Capture Hi–C, ChIP-seq, and ChIP–MS. Our results show that HMGN proteins occupy the A compartment in the 3D nucleus space. In particular, HMGN proteins occupy genomic regions involved in cell-type-specific long-range promoter–enhancer interactions. Interestingly, depletion of HMGN proteins in the three different cell types does not cause structural changes in higher order chromatin, i.e., in topologically associated domains (TADs) and in A/B compartment scores. Using ChIP-seq combined with mass spectrometry, we discovered protein partners that are directly associated with or neighbors of HMGNs on nucleosomes.

**Conclusions:**

We determined how HMGN chromatin architectural proteins are positioned within a 3D nucleus space, including the identification of their binding partners in mononucleosomes. Our research indicates that HMGN proteins localize to active chromatin compartments but do not have major effects on 3D higher order chromatin structure and that their binding to chromatin is not dependent on specific protein partners.

**Supplementary Information:**

The online version contains supplementary material available at 10.1186/s13072-022-00457-4.

## Introduction

Eukaryotic chromosomes are tightly organized into three-dimensional configurations within cell nuclei and play an essential role in DNA replication, transcription, and genome integrity [1]. The packaging of eukaryotic genomes starts with the double helical DNA, wrapping around a histone octamer to form the nucleosome structures, coiling into 30-nm-wide chromatin fibers, and further folding into 250-nm-wide chromatin fibers [2]. Due to dynamic long-range preferential interactions, chromosomes segregate into two forms of mutually excluded chromatin: A and B compartments. A compartments correspond to active transcription and open chromatin regions, while B compartments are compacted and enriched with repressive chromatin features [3, 4]. Recent advancements in chromosome conformation capture (3C) techniques, such as 5C and Hi–C, have led to a more comprehensive understanding of chromosome interaction maps over large regions. Topologically associating domains (TADs) are genomic regions with a higher frequency of intra-domain interactions and lower inter-domain contact frequencies. TAD sizes range between several hundred kilobases to a few megabases and act as functional insulation segments of the genome from neighboring regions [4–6]. TAD structures are strongly conserved in mammals and are maintained by the transcriptional repressor, CTCF, and cohesin, as well as by the cohesin loading and unloading factors, NIPBL and WAPL [7]. The combination of the A and B compartment distribution and TAD integrity is critical for gene regulation; perturbations of higher order chromatin structures lead to abnormal long-range interactions and nontarget gene activation [8]. Several other nuclear proteins have been found to be involved in higher order genome structural regulation, including YY1, PARP1, and PRDM5 [9–11]; however, how cellular nuclear proteins orchestrate and tightly regulate the mammalian genome structure remains unknown.

Chromatin architectural proteins directly interact with nucleosomes to modulate the chromatin status and genome stability [11]. Through interactions with nucleosomes, chromatin architectural proteins, such as heterochromatin-associated Protein 1 (HP1), human MeCP2 protein, and JUND, profoundly alter the intrinsic nature of chromatin fibers both in vitro or in vivo [12–14]. Further, linker histone H1 and high-mobility group N (HMGN) proteins represent two major families of chromatin architectural proteins that bind to nucleosomal core particles. Significantly histone H1 and HMGN proteins compete for nucleosomal core particles, leading to their different occupancies on chromatin [15–18]. Histone H1 proteins are enriched in constitutive heterochromatin, while HMGN proteins occupy active regulatory sites [19–21]. Two recent studies revealed that histone H1 protein depletion leads to chromatin decompaction, a B-to-A compartment switch, enhanced conferred germinal center B cell self-renewal, and de-repression of T cell activation genes [22, 23]. This raises a question as to whether HMGN proteins, as competitive partners of histone H1 proteins, can modulate 3D chromatin status.

In this study, we show how two major variants of HMGN proteins (HMGN1 and HMGN2) contribute to higher order chromatin structures in three different cell types derived from mice. By using high-resolution in situ Hi–C techniques, we demonstrate that HMGN proteins are enriched in the A compartment in the 3D nucleus. Next, by integrating Promoter Capture Hi–C data with ChIP-seq data, we find that HMGN proteins occupy genomic regions involved in cell-type-specific long-range promoter–enhancer interactions. Interestingly, HMGN protein depletion does not cause a notable alteration of higher order chromatin structure, e.g., there are no changes in TADs and the A/B compartment distribution. To characterize binding partners and proteins that co-occupy nucleosomes with HMGN proteins, we performed chromatin immunoprecipitation combined with mass spectrometry (ChIP–MS) using HMGN antibodies. We find that the protein partners of HMGNs have various functions in chromatin and DNA metabolism, broadly. Overall, our research provides new insights into the global organization of HMGNs in the nucleus.

## Results

### Nucleosomal-binding proteins HMGN1 and HMGN2 are located mainly in A compartments

**Fig. 1:**
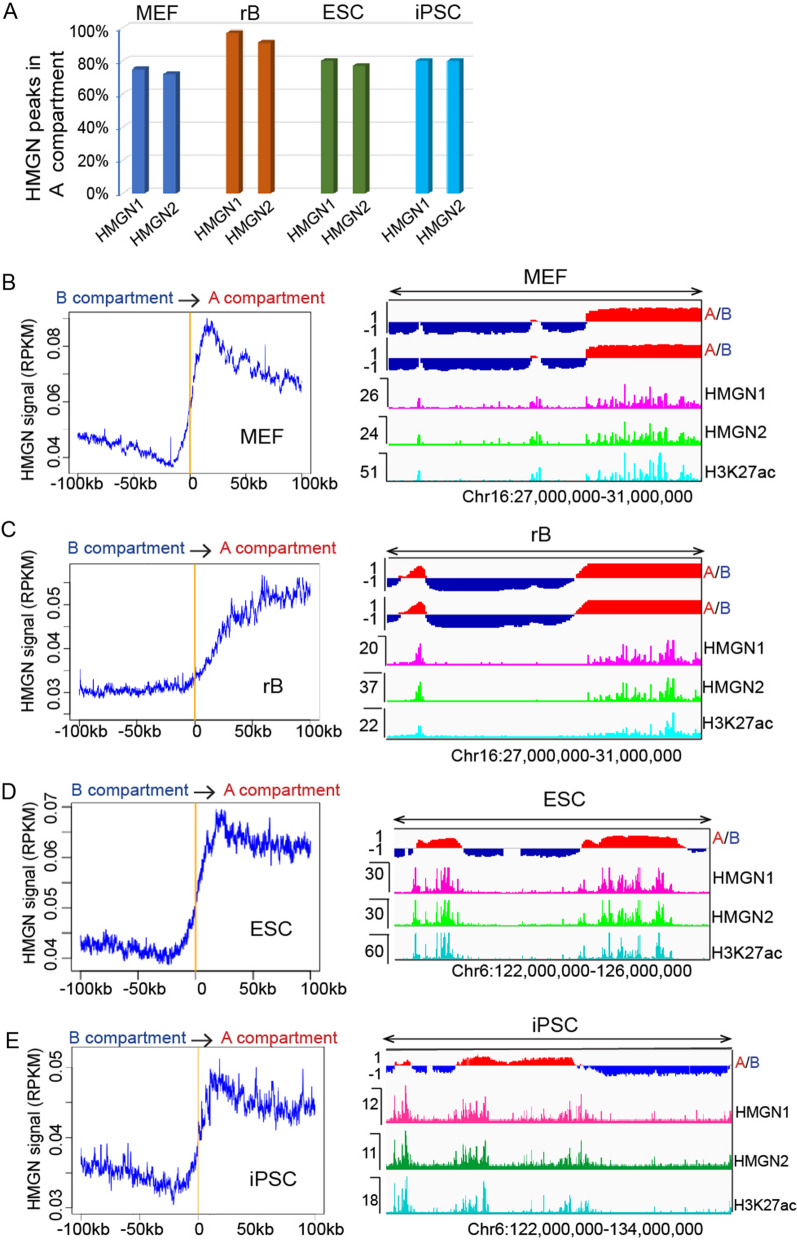
HMGN proteins (HMGN1 and HMGN2) are enriched at A compartments. **A** Percentage of HMGN1 and HMGN2 ChIP-seq peaks located in A compartment in MEFs, rBs, ESCs, and iPSCs. **B**–**E** Left panels: average HMGN1 signals in 200 kb windows across boundaries from B compartment to A compartment in MEF, rB, ESC and iPSC cells. **B**–**E** Right panels: individual examples of HMGN ChIP-seq signals enriched in A compartment and overlap with active enhancer maker H3K27ac. Snapshots are made from IGV genome browser

The mammalian genome is partitioned into A and B compartments, which correspond to transcriptionally active and silent chromatin, respectively [24]. Our previous studies showed that HMGN proteins bind to cell-type-specific regulatory sites, e.g., enhancers, super-enhancers, and assist in the regulation of gene transcription and cell identity [19, 20, 25]. However, whether HMGN protein enrichment in chromatin correlates with three-dimensional (3D) higher order chromatin structures is still unknown. In this study, we generated high-resolution in situ Hi–C data from three different cell types: mouse embryonic fibroblasts (MEFs), resting B (rB) cells, and induced pluripotent stem cells (iPSCs) [19]. We used Hi–C data of ESC cells from another study [26] and HMGN protein ChIP-seq data from our previous studies [19, 20]. We first identified and analyzed the A/B compartments in each of these cell types, using CScoretools [27]. Based on the A/B compartment identification results from CScoretools, the ratio of the genomic distance of compartment A to B is 4:5 in MEFs, 9.1:10 in resting B cells, 3:5 in ES cells, and 4:5 in iPSCs, suggesting that the total length of A and B compartments of the entire genome is similar in all of the cell types. Next, we integrated HMGN1/2 ChIP-seq data with Hi–C compartment analysis results. Our results show that HMGN proteins are located mainly within A compartments: in MEFs, about 75% of HMGN1 and HMGN2 peaks are enriched within the A compartment, and about 95%, 80%, and 80% HMGN peaks are in the A compartment in rBs, ES cells, and iPSCs, respectively (Fig. 1A). There is a sharp increase in HMGN signals across the boundaries between B and A compartments in all three cell types, as shown in Fig. 1B–E (left panels), which plot the average HMGN signals at a 200 kb window across all of the B → A boundaries. Our previous studies have shown that HMGN1 and HMGN2 signals are very similar across all the cell types. Therefore the results here are the average of HMGN1 and HMGN2. The B → A boundary regions of all four cell types exhibit stable and high mappability (75 bp read length and 2 mismatches allowed) of about 0.9 (Additional file 2: Fig. S2A), which excludes the possibility that the observed trend is influenced by any type of mapping bias due to sequence repeats. IGV genome browser snapshots (Fig. 1B–E, right panels) show individual examples of HMGN ChIP-seq signals highly enriched in the A compartment and that overlap with the active enhancer marker, H3K27ac. These results are consistent with our earlier studies that find that HMGN proteins are involved in active gene regulation [20, 28].

### Unaltered 3D chromatin structures upon HMGN protein depletion

**Fig. 2:**
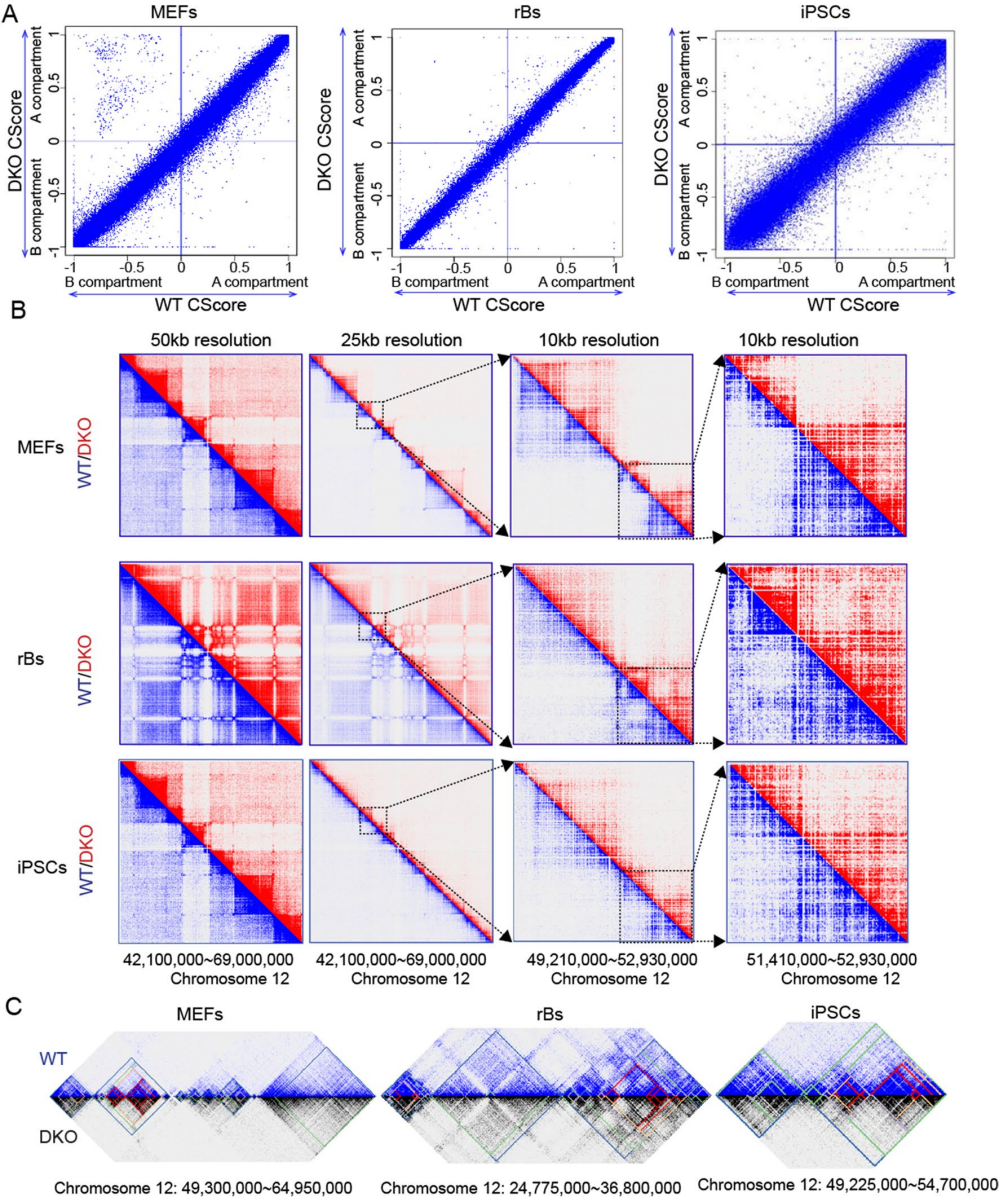
Unaltered 3D chromatin structure between WT and HMGN1/2 DKO cells. **A** Comparison of CScore of WT and DKO cells in MEF, rBs, and iPSCs. CScores are calculated at 25 kb resolution. **B** Examples of Juicebox illustration of similar Hi–C chromatin structure between WT and DKO at specific regions, at resolutions of 50 kb, 25 kb, and 10 kb in MEF, rB, and iPSCs. **C** Examples of similar TAD boundaries between WT and DKO in MEF, rB, and iPSCs. Hierarchical TADs are marked with lines of different colors

The eukaryotic genome is organized into distinct functional domains with different scales and compaction levels [2]. To understand the effect of HMGN proteins on higher order chromatin structures, we performed Hi–C experiments on wild-type (WT) and HMGN1/2 double knockout (DKO; *Hmgn1*^*−/−*^*; Hmgn2*^*−/−*^) MEF, rBs, and iPSCs. The WT and Hmgn DKO mice, and cells derived from these mice have been extensively characterized. DNA sequence analyses show the absence of the deleted genomic sequences, RNA sequence analyses show the absence of transcripts of the deleted exons, and Western analyses show that DKO cells do not express HMGN1 and HMGN2 proteins [19, 20, 25, 47]. Next, we generated Hi–C contact matrix maps, using the HiC-Pro pipeline [29], and visualized these data in Juicebox [29, 30]. We evaluated the reproducibility of replicates with the method of HiCRep [31]. The stratum adjusted correlation coefficient (SCC), an indicator of similarity levels between Hi–C interaction matrices, showed that the SCCs between any two of the WT replicates range from 0.985 to 0.995, and the SCCs between DKO replicates are at a similar level. Interestingly, the SCCs as compared with any WT and DKO samples also are at the same range, from 0.985 to 0.995. SCCs between samples from different cell types range from 0.5 to 0.62 (Additional file 2: Fig S1). These data suggest that the depletion of HMGN proteins does not significantly alter 3D chromatin contact matrixes. Next, we quantified A/B compartment differences between WT and DKO cells with CScoretools [27]. The output of CScoretools is a compartment score (C-score) that reflects the chance of a given genomic window being in the A or B compartment. The genome is divided into bins of 25 kb (bin sizes of 10 kb, 50 kb, and 100 kb generate similar results). A C-score ranging from − 1.0 to 1.0 is calculated for each bin. A positive value means A compartment while a negative value means B compartment (illustrated in Additional file 2: Fig S2A). The C-scores of WT cells is plotted against DKO cells for all bins (Fig. 2A). The CScore results showed that HMGN protein depletion has little effect on compartment scores, genome-wide, in all three cell types (Fig. 2A). Although there are some sites that switch from B compartment in WT to A compartment in DKO MEF cells (dots in the left top square), further examination of these dots revealed that they are from mainly two regions, at Chr4 and Chr13 (Additional file 2: Fig. S2C), and we did not find any correlation between these changes and gene expression. We noticed that the A/B compartment score differences between WT and DKO cells are greater in iPSCs than in MEFs and rBs, which is probably attributable to the fact that induced pluripotent stem cells have more open chromatin and are more sensitive to cellular nucleus status changes [32, 33]. Figure 2B further exemplifies the similarity of contact matrixes between WT and DKO cells at 50 kb, 25 kb, and 10 kb resolution.

TADs are considered the structural and functional units of mammalian genomes. TADs are characterized by a high frequency of intra-domain chromatin interactions but infrequent inter-domain chromatin interactions [34]. To investigate whether HMGN proteins affect TAD structures, we identified TADs in WT and DKO cells, using OnTAD [35]. Our results suggested that TAD structures remain intact in HMGN-depleted cells (Fig. 2C). Genome-wide, WT and DKO cells have a similar number of TADs with a comparable TAD size distribution (Additional file 2: Fig S3A, B). In summary, our results suggested that Hi–C contact matrices, A/B compartments, and TAD structures were largely unchanged upon HMGN protein depletion. Considering the highly conserved nature of TADs and higher order chromatin structures among different tissues or even species [36], it is not surprising that HMGN protein depletion causes no significant structural changes in higher order chromatin structures, as HMGs are not found in lower eukaryotes.

### HMGN proteins occupy promoter interaction regions that are highly enriched for *cis*-regulatory features

Enhancer–promoter interactions play a critical role in gene regulation. In particular, long-range *cis*-regulatory elements modulate target promoters through DNA looping and folding, a mechanism that bridges the distal enhancers to proximity to the target promoters [37]. Because our previous studies found that HMGN proteins bind to cell-type-specific regulatory sites [19], a related question is whether and how HMGN affects the spatial enhancer–promoter interactions in 3D nuclear space. To investigate this, we performed Promoter Capture Hi–C (PCHC) in WT and DKO MEFs, rBs, and induced pluripotent stem cells (iPSCs). The PCHC technique was developed to enrich promoter-containing ligation products from Hi–C libraries and to reduce the complexity of Hi–C libraries [38]. After mapping the readings with the HiCUP program [39], we identified significant promoter interaction regions (PIRs) by using the CHiCAGO pipeline with a threshold CHiCAGO score of ≥ 5 [40]. The numbers of significant PIRs from each experiment range from about 82,000 to 145,000, depending on the library sizes.

**Fig. 3:**
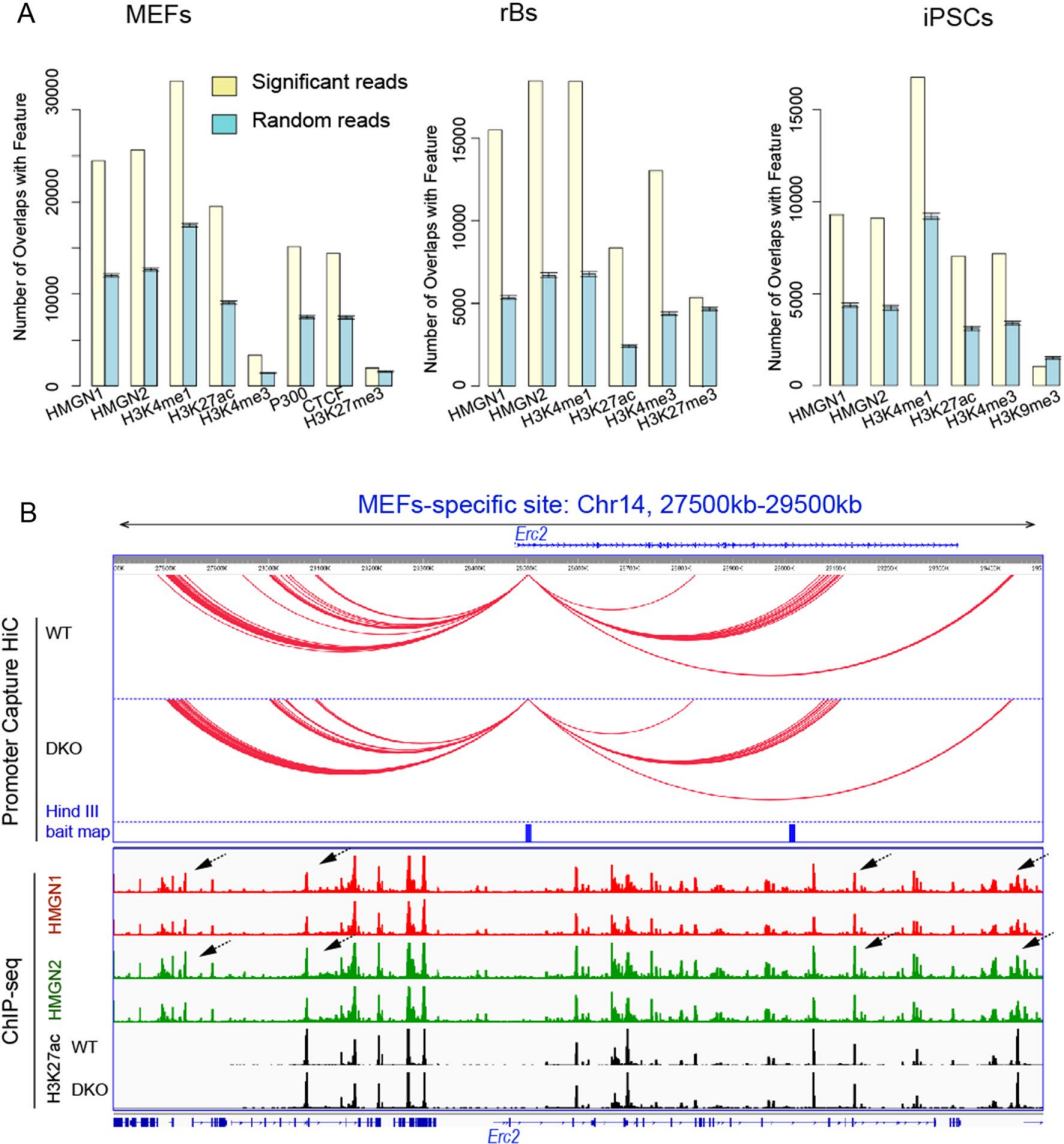
HMGN proteins bind to promoter interaction Regions (PIRs), which are highly enriched for cis-regulatory features involved in active transcription. **A** Chromatin features of promoter-interacting fragments detected with CHiCAGO. Yellow bars indicate overlaps of the genomic features with *cis*-interacting fragments within 1 Mb of promoter baits; blue bars indicate expected overlap values based on 100 random subsets of *HindIII* fragments. These subsets were selected to have a similar distribution of distances from gene promoters as the interacting fragments. Error bars represent 95% confidence intervals. Genomic features include ChIP-seq peaks of HMGN1/2, histone markers, CTCF, and p300. The difference between detected interactions and the expected value is significant for all genomic features with *p* value < 1e^−30^. The features involved in positive transcription regulation have a fold enrichment of ~ 2–3 while the repressive markers, H3K27me3 and H3K9me3, are either only slightly enriched or depleted. **B** Upper panel: snapshots of interactions with the Erc2 promoter identified with CHiCAGO in MEF cells. Lower panel: ChIP-seq signals of HMGN1, HMGN2 and H3K27ac in the same region. HMGN proteins specifically occupy at MEFs-specific site (Erc2) and its enhancers in MEF cells

The enrichment analysis of the CHiCAGO program showed that the identified PIRs are highly enriched in HMGN protein ChIP-seq signals and other genomic features involved in active transcription regulation, including histone markers H3K4me3, H3K27ac, and H3K4me1, and protein ChIP-seq signals for p300 and CTCF. The fold enrichment for these features ranges from 1.9 to threefold, For the repressive histone markers, H3K27me3 is only slightly enriched in the identified PIRs, with fold enrichment of 1.15 in MEF and 1.25 in rB cells. It might be related to the role of Polycomb proteins in nuclear architecture [41]. H3K9me3 is depleted to 71% in iPSCs (Fig. 3A). For example, Fig. 3B shows that the promoter of the MEF specific gene *Erc2* specifically contacts multiple genomic regions hundreds of kilobases away, both upstream and downstream, decorated with HMGN proteins and H3K27ac signals (Fig. 3B). There are, however, no significant similar contacts on those regions in rBs or iPSCs. Examples of cell-type-specific promoter–enhancer interactions and HMGN protein occupancy at PIRs in rBs and iPSCs are shown in Additional file 2: Fig. S4. Overall, our PCHC and ChIP-seq analysis reveal that HMGN proteins occupy cell-type-specific PIRs in the 3D genome.

We examined the statistically significant differential interactions in PCHC data between WT and DKO cells with Chicdiff [42]. We identified 131 differential interactions between MEF WT and DKO cells (Additional file 2: Fig. S5). These 131 sites, however, come from interacting regions with six gene promoters, for which there is no difference in gene expression levels between WT and DKO. We found no differential interactions in resting B cells or iPSCs. We thus determined that HMGN protein depletion has minor effects on promoter–enhancer interactions in the three different cell types, which is consistent with the Hi–C data analysis results that show that HMGN proteins do not directly regulate higher order chromatin structures.

### Proteomic profiling identifies proteins associated with HMGN proteins in chromatin

Previous examination of HMGN binding sites in various cells demonstrated the presence of an active chromatin signature at many of its binding sites. This signature is defined as a co-localization with high H3K27ac, H3K9ac, H3K4me1, and H3K4me3 histone markers, various nuclear factors, and increased chromatin accessibility [19, 20]. Preferential HMGN association with the A compartment in this work confirms the postulation that HMGNs contribute to various nuclear activities involved in transcription regulation. Next, we addressed the question of whether HMGN proteins have a preference to be juxtaposed to specific nuclear factors.

**Fig. 4:**
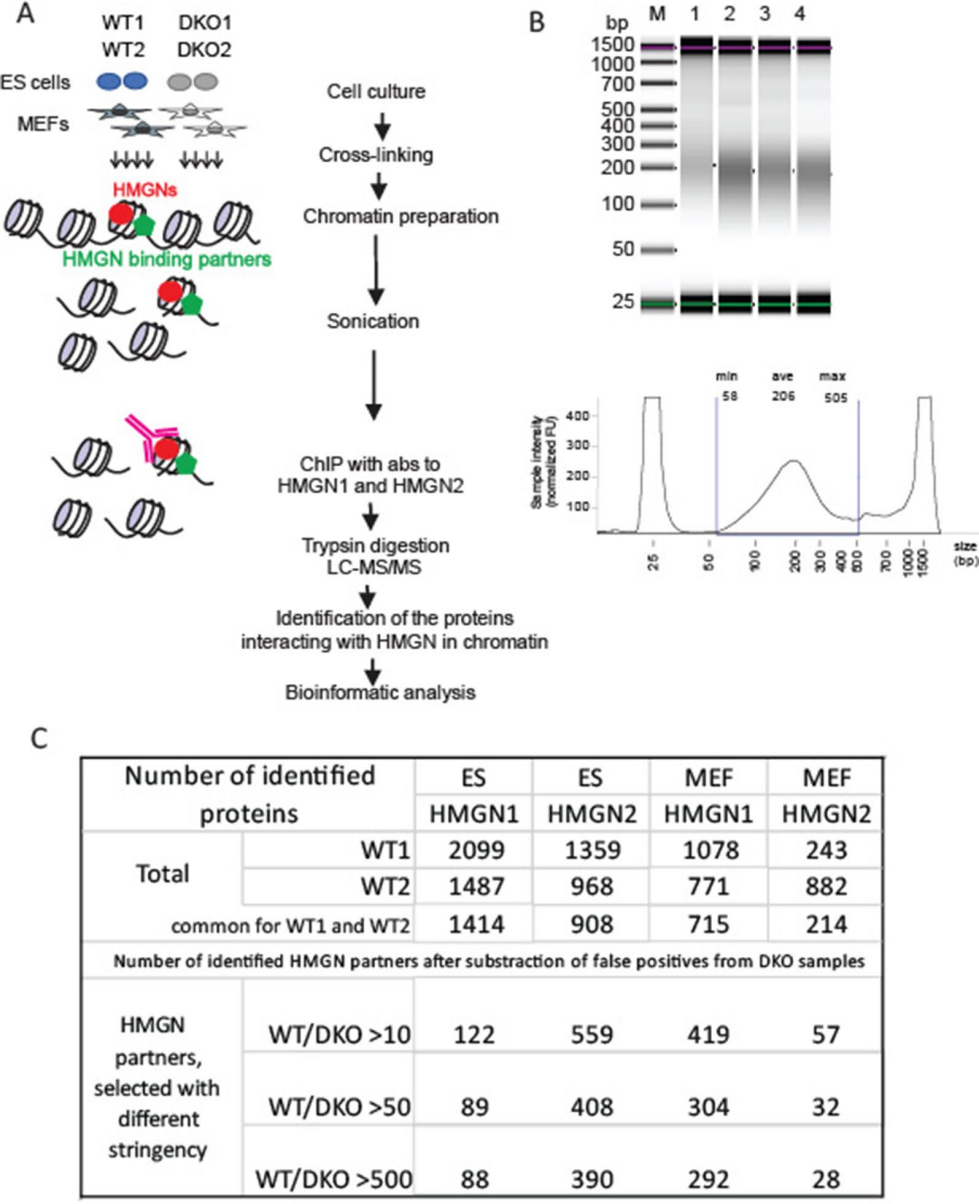
Chromatin profiling by ChIP–MS identifies proteins that reside nucleosome-long proximity to HMGN-occupied regions of chromatin. **A** Schematic overview of ChIP–MS assay. Mouse embryonic stem cells and MEF cells were cross-linked, and the chromatin was isolated, sonicated, and immunoprecipitated. DKO cells served as a negative control. Overall, there were 16 samples: two immunoprecipitations (HMGN1 and HMGN2), two cell types (ES and MEF), two genotypes (WT and DKO), and two biological replicates. Proteins, co-purified with HMGN and identified by UHPLC/MS/MS, represent HMGN binding partners. **B** ChIP DNA visualized by the Agilent TapeStation System after the optimized sonication step. More than 70% of total DNA fragments ranged from shorter than 505 bp to longer than 58 bp. **C** The table with the total number of identified proteins in individual samples. The number of proteins selected as HMGN binding partners with various (WT/DKO) cutoff limits in various cells

We performed chromatin immunoprecipitation combined with mass spectrometry (ChIP–MS) using HMGN antibodies in two cell types (ESCs and MEFs) derived from WT and DKO mice. We aimed to identify protein factors associated directly with or neighboring HMGN1 and HMGN2 proteins on nucleosomes [42, 43]. The protocol consists of the following steps: crosslinking, sonication, and immunoprecipitation with HMGN1 and HMGN2 antibodies, protease digestion, and LC–tandem MS (LC–MS/MS) analysis. This is followed by a computational murine database peptide search and protein identification (Fig. 4A). We reversed the cross-linked sonicated chromatin and checked DNA size distribution using TapeStation (Fig. 4B, C). Our results show that the fragmented DNA is normally distributed, with a peak of 180 bp, which ensures that protein factors identified by the ChIP–MS procedure are directly associated with or neighbors of HMGN proteins on the same nucleosome.

A successful ChIP–MS experiment generally results in the identification of 300–900 proteins, with 5–10% of these as specific binding partners [44]. All tests are abbreviated below as ES_N1, ES_N2, MEF_N1, and MEF_N2, and each test consisted of 4 samples: two WT and two DKO replicates. We observed 200–2000 protein factors per biological replicate (for a full list, see Additional file 1). All proteins identified by ChIP–MS analysis in DKO samples serve as false positive hits. Western analyses with antibodies to SMARCA5 and ATRX further verified that the ChIP MS data identified proteins that neighbor HMGN proteins. These proteins are detected in immunoprecipitates of WT cells but not in immunoprecipitates of the DKO control cells (Additional file 2: Fig. S6).

Two replicated average abundance values were calculated and served to establish a threshold measurement to identify true HMGN binding partners. The [WT/DKO] abundance ratio measures specific immunoprecipitation versus non-specific interactions. We defined proteins as specific HMGN binding partners if they have been preferentially identified in the target (WT) cells, either in negligible amounts or absent in the DKO cells. We considered that the criteria for a positive versus negative outcome would be the presence of protein in immunoprecipitated fractions of both WT biological replicates and that their average ((WT1 + WT2)/2) abundance was at least ten times higher than in DKO samples. We then identified 1157 factors with at least a tenfold difference in the abundance ratio, a measure for 1the amount of protein specifically immunoprecipitated, 833 factors at a 50-fold threshold, and 798 factors at a > 500-fold threshold (Fig. 4C). All subsequent analyses were conducted for proteins with WT/DKO ratios of > 10.

### HMGNs juxtapose proteins that execute various DNA- and chromatin-based activities

The lists of proteins—HMGN binding partners—in each of four groups of experiments are presented in Additional file 3: Table S1 (tabs indicate experiment groups). The proteins are shown with their average abundance value in HMGN-immunoprecipitated material. The abundance fluctuations may underlie distinct functional characteristics of the different cell types and various HMGNs and could depend on natural variation in their amount in a cell. Because HMGNs belong to a family of nucleosome-binding proteins, core and linker histones are expected among the proteins with the greatest abundance. We also noticed that there are no other distinct proteins or protein complexes that are significantly abundant.

Common for all samples are proteins with functions of epigenetic regulation and histone modification (see ES_N1, position #49—Jarid2; ES_N1, #37, and ES_N2, #120—Eed), cell cycle (ES_N1, #12—Aurkb; ES_N1, #79—Nsd2), transcriptional regulation (ES_N1, #44, and ES_N2, #173—H2ax, ES_N1, #34—Dmnt3a), chromatin remodeling (ES_N1, #21—Chd1; ES_N1, #49—Jarid2), DNA damage repair (ES_N1, #66—Mgmt; ES_N1, #59—Lig3). Some of the proteins are located in more than one list, including histone variant H2A.X. As expected, HMGN are detected among all binding partners (HMGN1: list ES_N1, #47, ES_N2, #192, and MEF_N1, #163; HMGN2: list ES_N2, #193, MEF_N1, #164).

To capture functional information on HMGN-binding partners, a comparative gene ontology (GO) Over-Representation Analysis (ORA) [1] was performed. ORA counts the number of proteins shared by an input set and each annotated set and applies a statistical test, such as the Fisher’s exact test, to calculate the statistical significance of the overlap between two sets. We first analyzed the protein sets—HMGN binding partners—using GO categories annotated by proteins that are related to various biological processes (PANTHER classification). A statistical significance threshold was selected as a conventional 5 × 10^–2^. HMGN binding partners from all four experiments (ES_N1, ES_N2, MEF_N1, and MEF_N2) yielded an over-representation of proteins from hundreds of various GO categories (Additional file 4: Table S2).

**Fig. 5:**
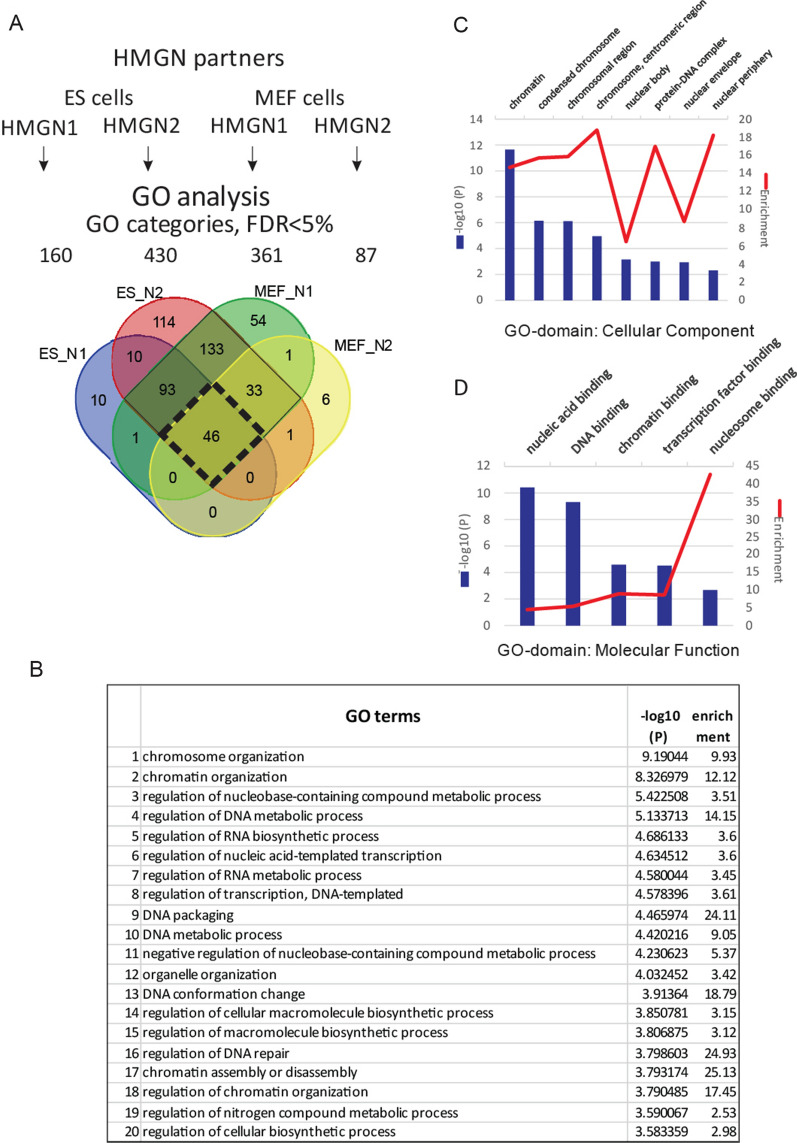
Gene ontology analysis for the HMGN binding partners identified by chromatin proteomic profiling. **A** Venn diagram that shows the overlap between overrepresented GO categories (FDR > 5%) for HMGN1 and HMGN2 binding partners in both cell types. The black diamond shows the GO categories (46) in which proteins are overrepresented among both HMGNs and both cell types. **B** List of the top 20 out of 46 GO categories over-represented among both HMGNs and both cell types. **C** Cellular component GO-category domain analysis of HMGN1 and HMGN2 binding partners in both cell types. Top eight categories ranked by − log_10_ (*P*) value are depicted. Bar graphs − log 10 (*P*), *P* is FDR-corrected *p* value, blue and line graphs (enrichment score), red. **D** Molecular function GO category domain analysis of HMGN1 and HMGN2 binding partners in both cell types. Top five categories ranked by − log_10_ (*P*) value are shown

To reveal common GO categories, we overlapped these in a Venn diagram (Fig. 5A). We observed a prominent similarity between HMGN-specific and cell-specific preferred GO-categories (318 common categories vs. 184 unique ones), and 46 were found to be over-represented in all four experimental groups (Fig. 5A, black box). To focus on top GO categories preferred by HMGN binding partners, a set of all HMGN binding partners was subjected to the ORA test; 70 GO categories with an FDR-adjusted *p* value of < 0.05 (Additional file 4: Table S2, Common GO tab) were found. Next, we ranked top 20 GO categories by − log_10_ (*P*), where (*P*) is an FDA-adjusted *p* value (Fig. 5B). Proteins involved in chromatin and chromosome organization (GO:0051276; GO:0006325) showed the highest − log_10_ (*P*) value, i.e. they are over-represented among HMGN binding partners.

To gain additional insight into functional characteristics of HMGN binding partners, we conducted the ORA test using two other GO categories: cellular component (Fig. 5C) and molecular function (Fig. 5D). The GO term “chromatin” has the highest − log_10_ (*P*) value (over 11), and the highest enrichment score (over 14). In the “Molecular Function” GO analysis the terms of DNA-, chromatin- and nucleosome-binding proteins (enrichment score of over 40) are among the highest HMGN binding partners.

Next, we compiled a list of the identified HMGN binding partners in all four experimental groups, belonging to “Chromatin organization” and other selective GO categories (Additional file 5: Table S3, “Chromatin organization,” “Chromatin remodeling,” “Histone modification,, “DNA repair,” “DNA packaging,” “Chromatin assembly,” and other tabs). Each category contains numerous HMGN colocalizing partners. Several proteins (e.g., Smarca5, Mecp2, and Kat6b) were present in several categories. We concluded that, by co-localizing with numerous different factors, HMGN proteins contribute to modulation of chromatin architecture and function.

## Discussion

Despite recent advances in our understanding of chromatin structures and functions, questions as to which architectural proteins contribute to chromosome stability, functional domain partitioning, and gene expression remain unanswered. Here we examine how the HMGN nucleosomal binding proteins position themselves in the 3D nucleus space and test whether they interact with unique epigenetic factors. Our research reveals that HMGN proteins preferentially occupy the A compartment in chromatin (Fig. 1). By analyzing Promoter Capture Hi–C data with HMGN protein and ChIP-seq data, we showed that HMGN proteins bind to genomic regions that are involved in cell-type-specific long-range promoter–enhancer interactions (Fig. 3; Additional file 2: Fig S2). In addition, through a ChIP–MS procedure we identified candidate HMGN binding partners and proteins that co-occupy nucleosome in the nucleus (Figs. 4, 5). Collectively, this study provides new information on the global chromatin organization of HMGN and on proteins that co-occupy nucleosomes with HMGN.

The eukaryotic genome is constructed in distinct domains to achieve genome stability and a tight regulation of gene expression.. Our previous studies demonstrated that HMGN proteins have an important regulatory function in the primary structure of chromatin. For example, we have shown that HMGN proteins modulate histone modification levels, chromatin accessibility, and transcription factor binding at lineage-specific regulatory sites [20, 25]. Through modulating the status of primary-structured chromatin, HMGN proteins fine tune many biological processes, such as B-cell activation, oligodendrocyte differentiation, and somatic cell reprogramming [19, 28, 43, 44]. Interestingly, our current study shows that HMGN protein depletion does not result in a notable alteration of higher order chromatin structural changes, e.g., TAD structures, A/B compartment scores, and long-range promoter–enhancer interactions in MEFs, B-cells, and iPSCs (Figs. 1, 2, 3). This finding may seem discrepant with our previous results, but, in fact, it should be attributed to the intrinsic nature of eukaryotic chromatin, which is tightly regulated at different structural levels [45]. A recent study shows that the degrading of Pol I–III proteins in mESCs does not change A/B compartment scores, local chromatin interactions, or TADs structures [46], which demonstrates that proteins that have a major role in active transcription and local chromatin status might have a very limited effect on higher order chromatin structures. Further, higher order chromatin structures, particularly TADs, are cell-type invariant and even conserved among different species [36]; therefore, we do not expect HMGN protein depletion to cause higher order structural changes in chromatin. We suggest that HMGN proteins modulate chromatin status mainly at its primary structural level for a more local regulatory effect, without altering the genome-wide 3D chromatin organizations.

HMGN proteins are non-histone chromatin architectural proteins that compete with histone H1 protein for binding to nucleosome core particles [15, 16, 18, 47]. Two recent studies show that histone H1 depletion in mice leads to a B-to-A compartment switch, chromatin decompaction, de-repression of T cell activation genes, and reactivation of early developmental genes key to germinal center B cells [22, 23]. Considering the competitive interactions between HMGN protein with histone H1 and their opposite effect on local chromatin structure observed in previous studies [15, 16, 48], it is tempting to assume that HMGN protein depletion could cause genome-wide compaction of chromatin and an A-to-B compartment switch. In fact, our current study shows that HMGN depletion causes few or no changes in A/B compartment strength. Histone H1 protein has a much greater abundance on nucleosome core particles than do HMGN proteins [15–17], suggesting that histone H1 protein depletion would cause a more significant effect on overall chromatin structures. Our previous studies have shown that HMGN proteins affect chromatin organization during the dynamic cell fate conversion process, such as cellular reprogramming and neuronal trans differentiation suggesting that HMGNs stabilize rather than determine chromatin organization at cell-type specific regulatory sites [19]. Future studies should address whether HMGN proteins modulate compartment establishment during trans differentiation and early development.

## Conclusions

Our results indicate that HMGN nucleosome binding proteins localize to the transcription active A compartment of chromatin. This finding is in agreement with our previous study which shows that HMGN proteins preferentially bind to acetylated nucleosomes in chromatin and stabilize cell-type-specific transcription programs. In addition, our ChIP–MS analysis indicates that HMGN proteins do not co-localize with a unique set of proteins in chromatin, suggesting that HMGN proteins are not recruited by specific regulatory factors. Finally, our findings suggest that HMGN proteins modulate accessibility to the active chromatin region thereby regulating cell type-specific gene expression.

## Methods

### Cell culture and medium

WT and DKO MEFs, naive B cells, ESCs, and iPSCs were prepared in our lab. The detailed cell culture conditions were described in our previous publications [19, 25, 28]. MEFs were prepared from E13.5 embryos and maintained in DMEM medium plus 10% FBS and 1% Pen Strep. Mouse ESCs and iPSCs were maintained on MEF feeder cells in the DMEM medium, which contained 15% FBS, 1% Pen Strep, Glutamax, Sodium Pyruvate, MEM Non-Essential Amino Acids, and 0.1 mM β-mercaptoethanol. For feeder-free culture, mouse ESCs and iPSCs were maintained in a knockout DMEM medium with 20% KOSR (Thermo Fisher Scientific, Cat# 10828028), 1% Pen Strep, Glutamax, Sodium Pyruvate, MEM Non-Essential Amino Acids 0.1 mM, β-mercaptoethanol, 1000  U mL^−1^, ESGRO^®^-2i Supplement Kit (1000×), and 1000  U mL^−1^ LIF (Millipore Sigma, ESG1121). Naive B cells were isolated from the spleens of 2-month-old WT and DKO male mice by immunomagnetic depletion, using anti-CD43 MicroBeads (Miltenyi Biotech). Purified B cells were cultured in RPMI 1640 media supplemented with 10% FBS and 1% Pen Strep, 1X GlutaMax™, 50 μM 2-mercaptoethanol.

### Chromatin immunoprecipitation followed by mass spectrometry (ChIP–MS) procedures

ChIP–MS experiments were performed by following the published protocol with minor modifications [49]. We prepared two replicates for each cell type, and 30 million cells were used for each replicate reaction. Briefly, cells were cross-linked using 1% (vol/vol) formaldehyde, and the reaction was quenched by 0.125 M glycine. Cell lysates were prepared by using an LB1, LB2, and LB3 buffer. The purified cell nuclei were sonicated by using a Diagenode sonicator (30 s on and 30 s off, 12 cycles). The sonication efficiency and total chromatin were verified by running the sonicated DNA on a TapeStation. The fragmented DNA size ranged between 200 and 500 bp.

For each reaction, 10 µg of HMGN1 antibody (Bustin lab) or HMGN2 antibody (Cell Signaling Technology, #9437) were conjugated to Protein A beads (Thermo Fisher Scientific, #10006D), followed by overnight incubation with sonicated chromatin at 4 °C. The next day, the immunoprecipitated complex was washed 10 times by a RIPA buffer at 4 °C, followed by washing two times in 1 mL of cold 100 mM ammonium hydrogen carbonate (AMBIC) solution.

### Enzymatic digestion and LC–MS/MS procedures

Samples were processed and in-solution digested with trypsin using S traps (Protifi), following the manufacturer’s instructions. Briefly, proteins were denatured in 5% sodium dodecyl sulfate (SDS) and 50 mM triethylammonium bicarbonate (TEAB) pH 8.5. They were next reduced with 5 mM tris(2-carboxyethyl)phosphine (TCEP) and alkylated with 20 mM iodoacetamide. The proteins were acidified to a final concentration of 2.5% phosphoric acid and diluted into 100 mM TEAB pH 7.55 in 90% methanol. They were loaded onto the S-traps, washed four times with 100 mM TEAB pH 7.55 in 90% methanol, and digested with trypsin overnight at 37 °C. Peptides were eluted from the S-trap, using 50 mM TEAB pH 8.5, 0.2% formic acid in water, and 50% acetonitrile in water. These elution fractions were pooled and dried by lyophilization.

Dried peptides were resuspended in 5% acetonitrile, 0.05% TFA in water for mass spectrometry analysis. The peptides were separated on a 75 µm × 15 cm, 3 µm Acclaim PepMap reverse phase column (Thermo Fisher Scientific) at 300 nL/min, using an UltiMate 3000 RSLCnano HPLC (Thermo Fisher Scientific), through a two-step linear gradient from 96% mobile phase A (0.1% formic acid in water) to 35% mobile phase B (0.1% formic acid in acetonitrile) over 120 min and 35% mobile phase B to 55% mobile phase B over 10 min. The peptides were analyzed using an Exploris480 Orbitrap mass spectrometer (Thermo Fisher Scientific), with parent full-scan mass spectra acquired at 120,000 FWHM resolution and product ion spectra at 15,000 resolution.

Proteome Discoverer 2.4 (Thermo Fisher Scientific) was used to search the data against the murine database from UNIPROT, using Sequest HT. The search was limited to tryptic peptides, with maximally two missed cleavages allowed. Cysteine carbamidomethylation was set as a fixed modification, with methionine oxidation as a variable modification. The precursor mass tolerance was 10 ppm, and the fragment mass tolerance was 0.02 Da. The Percolator node was used to score and rank peptide matches, using a 1% false discovery rate. Label-free quantitation of extracted ion chromatograms from MS1 spectra was performed using the Minora node in Proteome Discoverer.

### ChIP–MS data analysis

Protein quantitative analysis was performed in Microsoft Excel. For all samples, the proteins were evaluated according to their abundance in the WT vs. DKO samples. The algorithm for awarding a title of HMGN binding partner was as follows: Abundance of any protein is WT1 ≠ 0, WT2 ≠ 0, and WT_ave_/DKO_ave_ ≥ 10, where WT_ave_ and DKO_ave_ mean averages of two biological replicates. Protein sets are enlisted in Additional file 3: Table S1. GO enrichment analysis (over-representation test, ORA) was performed with various GO terms, using available annotations. A full-results table (Additional file 1) was saved with significant shared GO terms (or parents of GO terms), background frequencies, sample frequencies, expected *p* values, an indication of over/underrepresentation for each term, and *p* value). An FDA-adjusted *p* value was used to calculate − log_10_ (*P*).

### Hi–C and promoter capture HiC library preparation, DNA library sequencing

HiC and Promoter Capture HiC libraries were prepared by following the published protocol with minor modifications [38]. First, cells were cross-linked, using 1% formaldehyde for 10 min and quenched by 0.125 M glycine for 5 min at room temperature. Five million cells were used for each HiC reaction. Cells were lysed in 10 mL of a freshly prepared ice-cold lysis buffer (10 mM Tris–HCl pH 8, 0.2% (vol/vol) Igepal CA-630, 10 mM NaCl, and one tablet protease inhibitor cocktail) on ice for 15 min, followed by centrifuging at 760×*g* for 5 min at 4 °C to remove the supernatant. Next, the nuclei pellet was washed in 1.25X NEB buffer2 (NEB, B7002S) and resuspended in 358 µL 1.25 × NEB buffer2, followed by the addition of 11 µL of 10% (wt/vol) SDS and shaking at 950 rpm for 30 min at 37 °C. The SDS was quenched by adding 10% Triton X-100 (vol/vol) per aliquot and shaking at 950 rpm at 37 °C for 15 min. The nuclei were digested by using 12 µL of 100 U/µL Hind III (NEB, R0104M) at 37 °C overnight with constant shaking. The next morning, the Hind III digested overhangs were repaired by 6.1 µL 10 × NEB buffer2, 25 μL H_2_O, 15.3 μL of 1 mM biotin-14-dATP (Jena Bioscience, NU-835-Bio14-L), 1.56 μL of 10 mM dCTP, 1.56 μL of 10 mM dGTP, 1.56 μL of 10 mM dTTP, and 10.2 μL of 5 U/µL DNA Polymerase I, Large (Klenow) Fragment (NEB, M0210L) and incubated at 37 °C for 1 h. In-nucleus ligation was performed by adding 102 μL 10 × T4 DNA ligase buffer, 10.2 μL BSA(NEB, B9000S), 350.9 μL H_2_O, and 25.5 μL 1 U/μL T4 DNA ligase (Thermo Fisher Scientific, 15224017), followed by incubation at 16° for 4 h in a thermomixer. The nuclei samples were centrifuged at 2500*g* for 5 min and resuspended in a 300 μL de-crosslinking buffer (10 mM Tris–HCl, 0.5 M NaCl, 1% SDS), followed by adding 5 μL 10 mg/mL RNase A (Thermo Fisher Scientific, EN0531) RNase A at 37 °C 30 min, followed by adding 20 μL 20 mg/mL Proteinase K (Gold Bio, P-480-SL2) and incubation at 55 °C for 1 h and 68 °C overnight. The following morning, 825 μL of pure ethanol and 50 μL of 3 M sodium acetate (pH 5.2) were added to each reaction, followed by incubation at − 80 °C for 15 min. Tubes were centrifuged at a maximum speed for 15 min to precipitate DNA. The DNA pellet was washed twice with 70% ethanol and air dried for 5 min at room temperature, and resuspended in 130 μL of 10 mM Tris–HCl. To fragment DNA, DNA samples were transferred to a micro-TUBE AFA fiber Pre-Slit Snap-Cap (Covaris, 520045) and sonicated to 300–500 bp using an ME220 Covaris sonicator with the following parameter setups: Peak Incident Power 50 W, Duty Factor 20%, Cycles per Burst 200, Treatment time 80 s. The sonicated DNA was double-sided selected by using AMPure XP beads (Beckman Coulter, A63881). First, 120 μL of beads were added to each reaction (ratio of AMPure beads to DNA: 0.6–1) and incubated for 5 min. By using a magnetic separation stand, the clear supernatant was transferred to a fresh tube. Next, 30 μL AMPure XP beads were added to the clear supernatant and incubated for 5 min. Beads were separated on a magnet stand again and washed two times with 70% ethanol. Beads were dried at 37 °C in a thermomixer for 3 min, and the DNA was eluted in a 300 μL 1 × Tris buffer. The fragment size distribution of DNA was verified by running TapeStation.

For Biotin/Streptavidin pull-down of the Hi–C ligation products, 150 μL 10 mg/mL of Dynabeads™ MyOne™ Streptavidin C1 (ThermoFisher Scientific, 65001) were washed twice in a 500 μL 1 × Tween Washing Buffer (5 mM Tris–HCl pH 8.0; 0.5 mM EDTA; 1 M NaCl) and re-suspended in a 300 μL 2 × binding buffer (10 mM Tris–HCl pH 8.0; 1 mM EDTA; 2 M NaCl), followed by adding to the 300 μL DNA sample and incubating at room temperature for 15 min. Next, the streptavidin beads were washed twice by using a 600 μL 1 × Tween Washing Buffer at 55 °C for 2 min with constant shaking. For the end repair and removal of biotin at the non-ligated DNA ends, a 88 μL 1 × NEB T4 DNA ligase buffer with 1 mM ATP (B0202S), 1 μL of 10 mM dNTP (ThermoFisher, R0192), 5 μL of 10 U/μL NEB T4 PNK (m0210L), 4 μL of 3 U/μL NEB T4 DNA polymerase I(M0203L), and 1 μL of 5 U/μL NEB DNA polymerase I Large Klenow (M0210L) were added to the beads and incubated at room temperature for 1 h, followed by washing twice, using a 600 μL 1 × Tween Washing Buffer at 55 °C for 2 min with constant shaking and one-time washing, using a 100 μL 1 × NEB buffer 2. For the dATP tailing, a 90 μL 1 × NEB buffer 2, 5 μL 10 mM dATP, and 5 μL 5 U/μL NEB Klenow exo- (M0212L) were added to the beads and incubated at 37 °C for 30 min. Next, the beads were washed twice, using a 600 μL 1 × Tween Washing Buffer at 55 °C for 2 min with constant shaking and a 200 μL 1 × NEB quick ligation buffer and re-suspended in a 60 μL 1 × NEB quick ligation buffer. The NEB Quick Ligation™ Kit (M2200L) and NEBNext^®^ Multiplex Oligos for Illumina^®^ (Index Primers Set 1) (E7335S) were used for adaptors ligation. In addition, a 2 μL quick ligase and 3 μL adaptor were added to the beads and incubated at 25 °C for 15 min, followed by a 3 μL USER enzyme treatment at 37 °C for 15 min. Next, beads were washed twice, using a 600 μL 1 × Tween Washing Buffer at 55 °C for 2 min with constant shaking and a 200 μL 1 × NEB buffer2 and re-suspended in a 60 μL 1 × NEB buffer 2. The HiC libraries were amplified by using the NEB Phusion High-Fidelity PCR kit (E0553L), using the following PCR conditions: 30 s at 98 °C; seven cycles of 10 s at 98 C, 30 s at 65 °C, 30 s at 72 °C; followed by a 7-min extension at 72 °C and held at 4 °C. Amplified HiC libraries were size selected by using AMPure beads at a ratio of 0.8 × and eluted in a 60 μL 1 × Tris buffer. The HiC library size distribution was evaluated, using TapeStation software, and the HiC library concentration was first estimated by using the Qubit 4 Fluorometer, followed by quantification using the KAPA Library QANT Kit (Roche, E7770S).

To enrich promoter-associated Hi–C ligation fragments, we performed Promoter Capture experiments, using SureSelectXT Custom 3–5.9 Mb (Agilent, 5190-4831) and SSEL TE Reagent Kit, ILM PE FULL Adaptor (Agilent, 5190-4831). In addition, 1000 ng of HiC DNA were aired dried on a Speed Vacuum Concentrator (Thermo Fisher Scientific), followed by hybrid in-solution capture. Dynabeads™ MyOne™ Streptavidin C1 beads were used to pull down the HiC fragment associated with promoters. The Promoter Capture HiC libraries were amplified, using the NEBNext^®^ Multiplex Oligos for Illumina and NEB Phusion High-Fidelity PCR kit, followed by AMPure beads size selection at the ratio of 0.8. The Promoter Capture HiC library size distribution was evaluated by using TapeStation software, and the library concentration was first estimated by using the Qubit 4 Fluorometer, followed by quantification using the KAPA Library QANT Kit (Roche, E7770S). Both the HiC and Promoter Capture HiC libraries were pair-ended sequenced (2 × 50 bp) on Novaseq S2 flow cells.

### Hi–C and promoter capture Hi–C data analysis

Hi–C readings from separate lines were concatenated, aligned to the mouse genome (mm10), and processed with HiC-Pro (v.3.0.0) [29], using default parameters and genomic bin sizes of 25 and 100 kb. The valid pairs from HiC-Pro mapping were used to generate Hi–C files for Juicebox visualization with juicer tools (juicer_tools_1.22.01.jar). We used HiCRep to assess the reproducibility of our Hi–C replicates. Intrachromosomal contacts were extracted for chromosome 1, using binned readings at 25-kb resolution. We then used these contacts to calculate the stratum-adjusted SCC between WT and DKO replicates. We have three replicates of each sample. The SCCs between any two of the WT replicates range from 0.985 to 0.995, and the SCCs between DKO replicates are similar.

Replicates of each genotype were used as input for multiHiCcompare (github.com/dozmorovlab/multiHiCcompare) to determine statistically significant differences in contact frequencies between genotypes. Replicates were pooled for A/B compartment analysis with CScoreTool (GitHub—scoutzxb/CscoreTool). C-scores of 25-kb and 100-kb bins were calculated from the intrachromosomal contact matrices and compared between WT and DKO samples. Analyses of TADs were performed using OnTAD (GitHub—anlin00007/OnTAD: An Optimized Nested TAD caller for Hi–C data). Hi–C contact matrices were calculated for all autosomes with 25,000-bp resolution matrices (-penalty 0.1 -minsz 3 -maxsz 200 -lsize 5 ldiff = 1.96).

For each cell type, we prepared and sequenced two Promoter Capture Hi–C libraries. Promoter capture Hi–C data analysis using previously published software. Interaction confidence scores were computed using the CHiCAGO pipeline[40]. Interactions with a CHiCAGO score of > 5 were considered high-confidence interactions. The Chicdiff package was used to identify differential interactions between WT and DKO cells [42].

## Supplementary Information

**Additional file 1**: Excel spreadsheet ChIP–MS raw data for all 16 samples.

**Additional file 2**: **Fig. S1**. Stratum correlation coefficients (SCCs) between Hi–C data samples. There are two or three replicates for WT and DKO MEF and rBs. There is one sample for WT and DKO iPSCs. The SCC among all WT and DKO replicates ranges from 0.985 to 0.995 for MEF or rBs. The SCC between WT and DKO iPSC is 0.977. The SCCs between samples from different cell types are about 0.5 (MEF vs. rB or iPSC vs. rB) and 0.62 (MEF vs. iPSC). **Fig. S2** .A) 75 bp mappability scores (2 mismatches allowed) was computed for the mm10 genome using GenMap (https://github.com/cpockrandt/genmap). The average mappability across all the compartment B- > A boundaries are calculated for MEF, rB, ESC and iPS cell types. B) An illustration of how, Fig. 2A, was made. The whole genome is divided into bins of 25 kb. A C-score ranging from -1.0 to 1.0 is calculated for each bin. The C-scores of WT cells is plotted against DKO cells for all the bins. C) Two genomic regions, one at chr4 and the other at chr13, show a switch from B compartment to A compartment (marked with red frames) upon depletion of HMGN proteins. No correlations with gene expression was found. The RNA-seq data of WT and DKO MEFs is shown in the bottom two rows. **Fig. S3**. Comparison of TAD-calling results between WT and DKO cells. (A) Proportion of TADs according to the size range in three different cell types. (B) TADs size distribution in three different cell types. **Fig. S4**. Snapshots of cell type-specific interactions identified with CHiCAGO in rBs, and iPSCs, lined with ChIP-seq signals of HMGN1, HMGN2 and H3K27ac in the same regions, similar to Fig. 3. (A) Genomic regions around the promoter of an rB-specific gene Foxp1. (B) Genomic regions around the promoter of a pluripotency-specific gene Nanog. **Fig. S5**. Regions of differential interactions between MEF WT and DKO cells identified with Chicdiff. A total of 131 differential interactions are from regions related to six genes: U1.119 on Chr16, Fgfbp3, Btaf1, U6.858, Ide, and Kif11 on Chr19. For each gene, significant interactions detected with CHiCAGO are represented with color-coded dots (blue: 3 < Chicago score ≤ 5; red: Chicago score > 5). The upper panel contains the WT MEF cells and lower panel contains the DKO cells. Between the upper and lower panels are differentially interacting regions detected by Chicdiff, depicted as color-coded blocks with the color as representing a *p* value range. Interactions beyond 1 Mb each way were cropped. **Fig. S6**: Validation of the ChIP–MS procedure by ChIP-Western analysis. Western blot analyses of Smarca5, Atrx and Hspa8 proteins in immunoprecipitated chromatin fractions. The proteins from 0.1% input chromatin and the proteins, purified by ChIP with antibodies to either HMGN1 (A) or HMGN2 (B) was loaded on the gel, and probed with mouse Smarca5 (ThermoFisher Scientific MA3-055), Atrx (ThermoFisher Scientific CL0537) and Hspa8 (ThermoFisher Scientific 13D3) antibodies. Input and ChIP preparations are indicated at the top. The antibodies used for the Western blot analyses are in the middle. The abundance values for indicated proteins (from Additional file 1) were used to calculate a standardized Z-score. Obtained Z-scores were further color-coded.

**Additional file 3: Table S1**. Proteins—HMGN1 and HMGN2 partners in both cell types.

**Additional file 4: Table S2**. GO analysis of HMGN1 and HMGN2 partners in both cell types.

**Additional file 5: Table S3**. Proteins—HMGN1 and HMGN2 partners in top GO categories.

## Data Availability

The HiC and Promoter Capture Hi–C data reported in this paper are available with the accession numbers: BioProject: PRJNA825595 (https://www.ncbi.nlm.nih.gov/bioproject/?term=PRJNA825595). All other relevant data that support the key findings of this study are available within the article and its Additional files or from the corresponding author upon reasonable request.
