# Supplementary figures and images for: Multiple epigenetic factors co-localize with HMGN proteins in A-compartment chromatin
Source: Epigenetics Chromatin. 2022 Jun 27;15:23. doi: 10.1186/s13072-022-00457-4 (PMC9235084; doi:10.1186/s13072-022-00457-4)

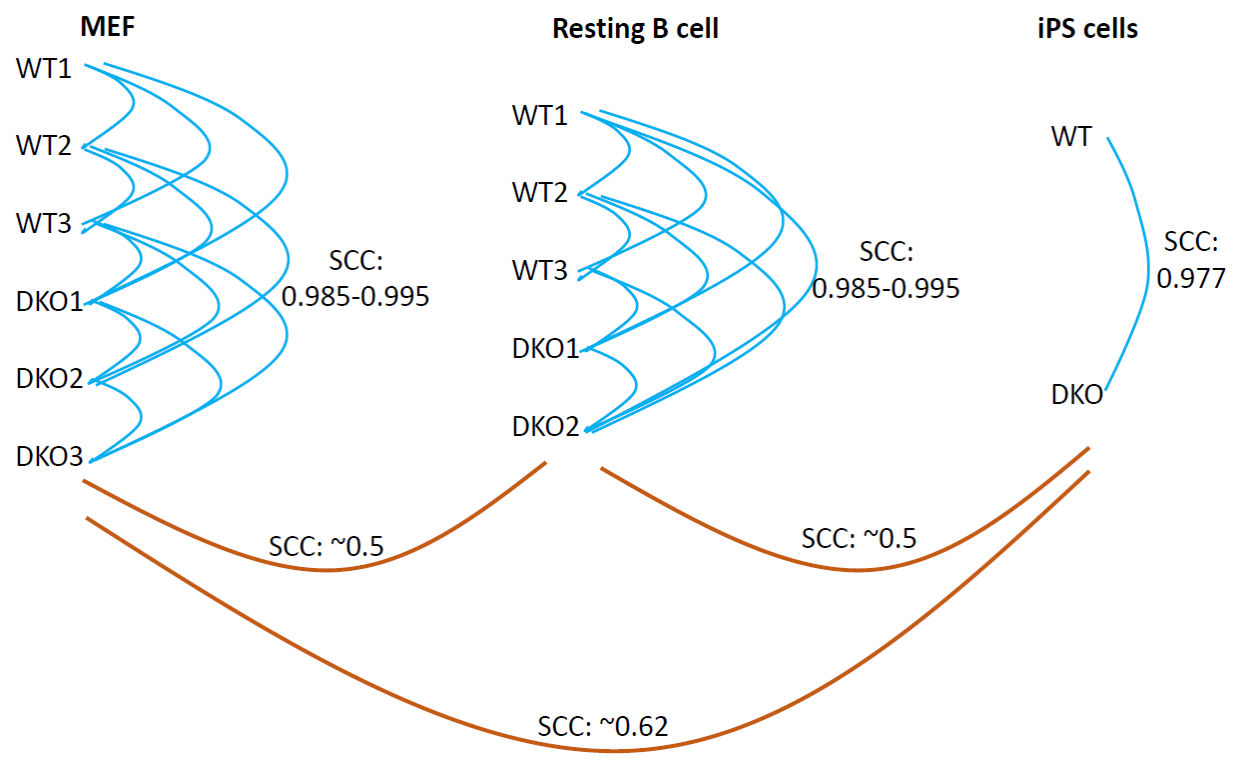

Figure S1

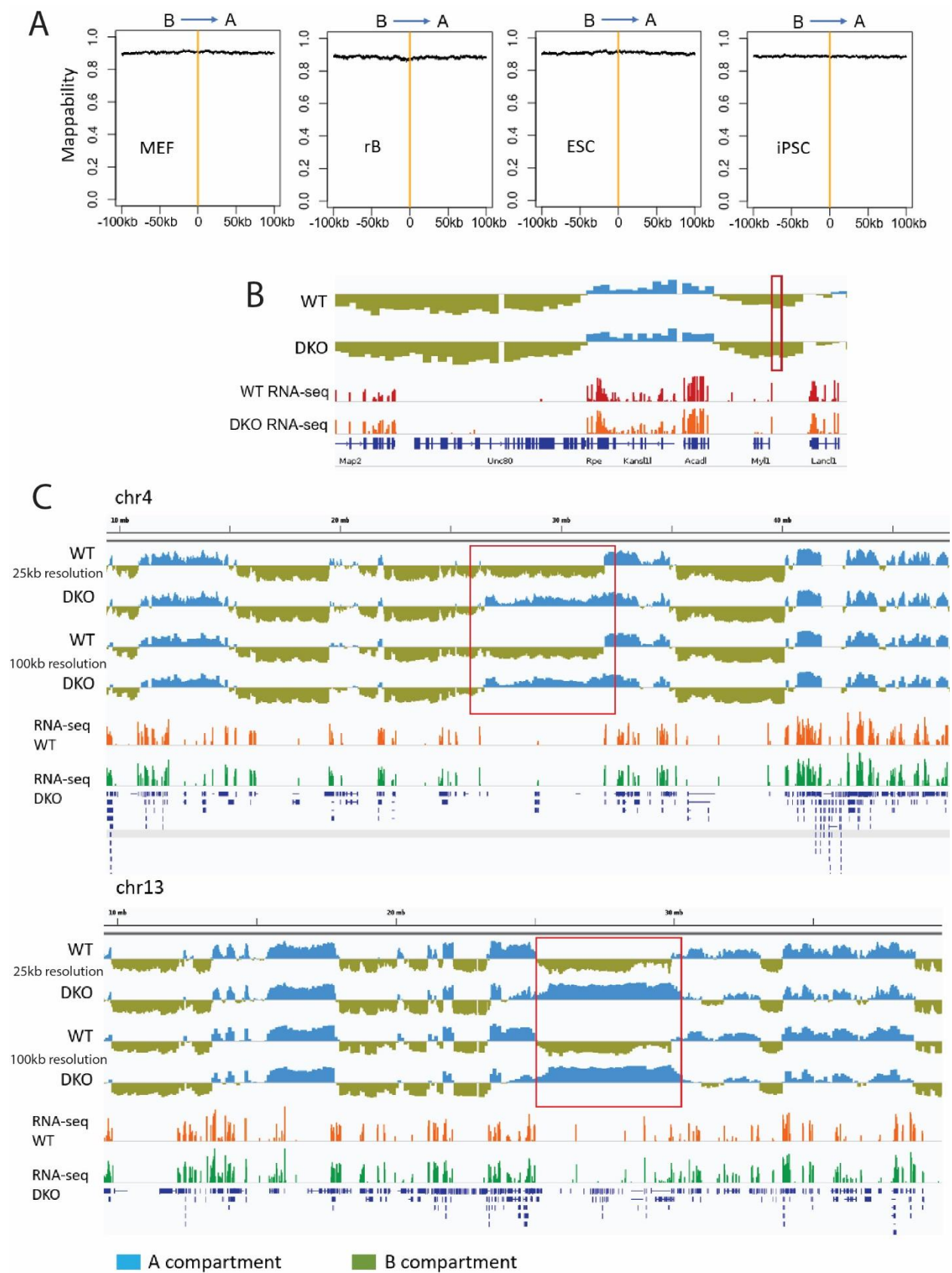

Figure S2

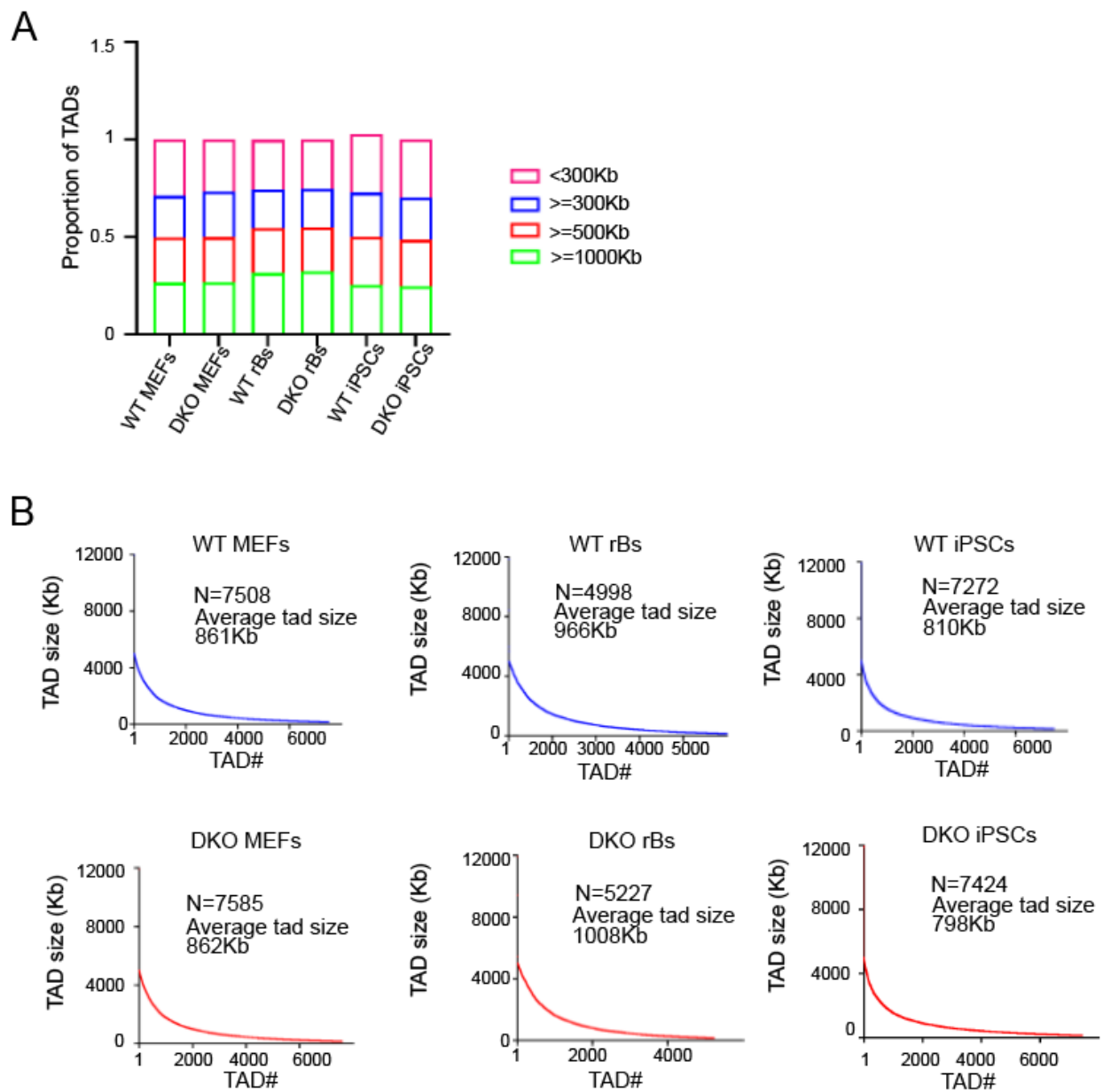

Figure S3



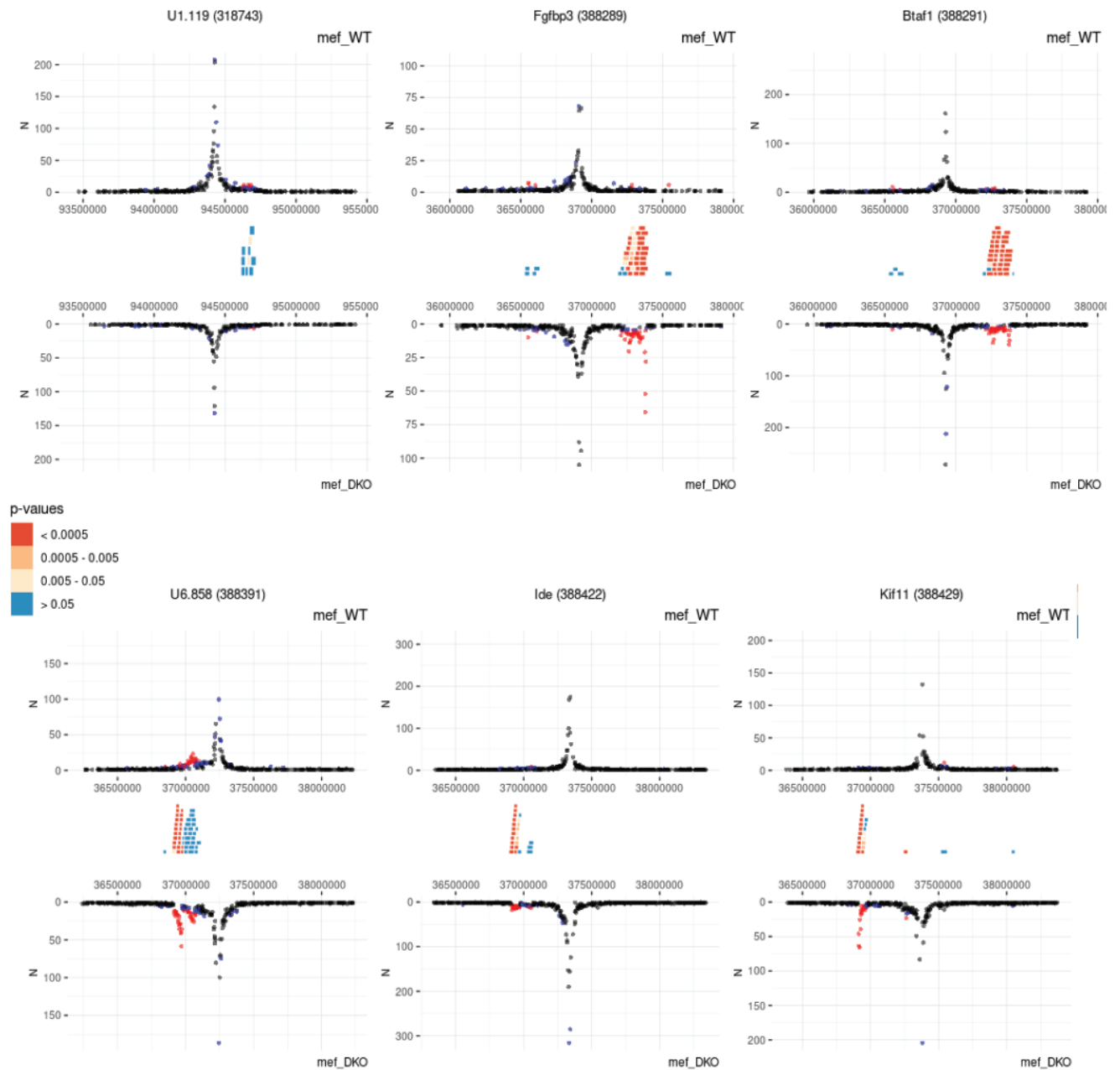

Figure S5

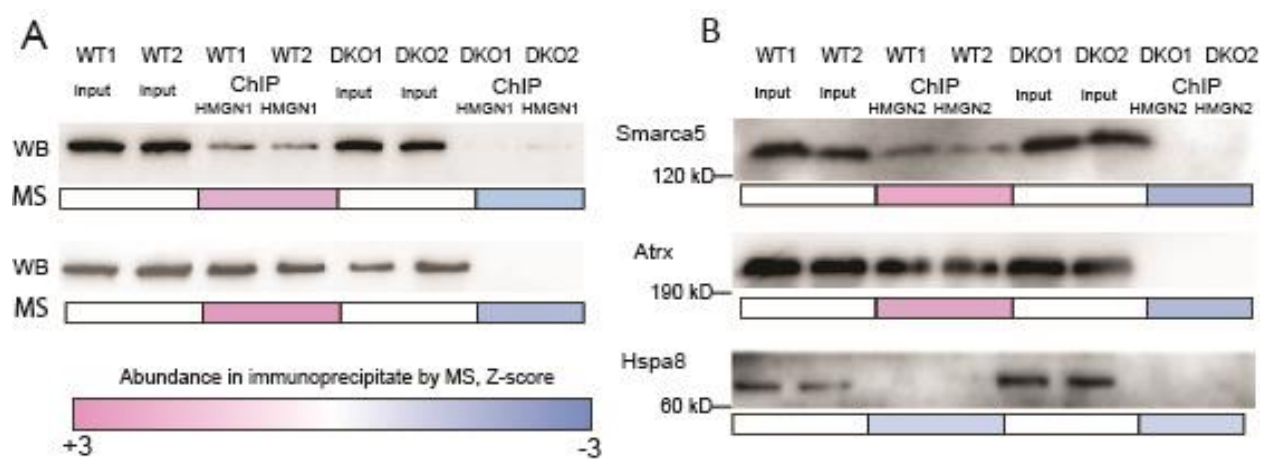

Figure S6

Supplement: Supplementary file 2 — Additional file 2: Fig. S1. Stratum correlation coefficients (SCCs) between Hi–C data samples. There are two or three replicates for WT and DKO MEF and rBs. There is one sample for WT and DKO iPSCs. The SCC among all WT and DKO replicates ranges from 0.985 to 0.995 for MEF or rBs. The SCC between WT and DKO iPSC is 0.977. The SCCs between samples from different cell types are about 0.5 (MEF vs. rB or iPSC vs. rB) and 0.62 (MEF vs. iPSC). Fig. S2 .A) 75 bp mappability scores (2 mismatches allowed) was computed for the mm10 genome using GenMap (https://github.com/cpockrandt/genmap). The average mappability across all the compartment B- > A boundaries are calculated for MEF, rB, ESC and iPS cell types. B) An illustration of how, Fig. 2A, was made. The whole genome is divided into bins of 25 kb. A C-score ranging from -1.0 to 1.0 is calculated for each bin. The C-scores of WT cells is plotted against DKO cells for all the bins. C) Two genomic regions, one at chr4 and the other at chr13, show a switch from B compartment to A compartment (marked with red frames) upon depletion of HMGN proteins. No correlations with gene expression was found. The RNA-seq data of WT and DKO MEFs is shown in the bottom two rows. Fig. S3. Comparison of TAD-calling results between WT and DKO cells. (A) Proportion of TADs according to the size range in three different cell types. (B) TADs size distribution in three different cell types. Fig. S4. Snapshots of cell type-specific interactions identified with CHiCAGO in rBs, and iPSCs, lined with ChIP-seq signals of HMGN1, HMGN2 and H3K27ac in the same regions, similar to Fig. 3. (A) Genomic regions around the promoter of an rB-specific gene Foxp1. (B) Genomic regions around the promoter of a pluripotency-specific gene Nanog. Fig. S5. Regions of differential interactions between MEF WT and DKO cells identified with Chicdiff. A total of 131 differential interactions are from regions related to six genes: U1.119 on Chr16, Fgfbp3, Btaf1, U6.858, Ide, [file 13072_2022_457_MOESM2_ESM.pdf]
